# A tied Fermi liquid to Luttinger liquid model for nonlinear transport in conducting polymers

**DOI:** 10.1038/s41467-020-20238-5

**Published:** 2021-01-04

**Authors:** Jiawei Wang, Jiebin Niu, Bin Shao, Guanhua Yang, Congyan Lu, Mengmeng Li, Zheng Zhou, Xichen Chuai, Jiezhi Chen, Nianduan Lu, Bing Huang, Yeliang Wang, Ling Li, Ming Liu

**Affiliations:** 1 Key Laboratory of Microelectronic Devices & Integrated Technology, Institute of Microelectronics, Chinese Academy of Sciences, Beijing, 100029 China; 2 Shenzhen JL Computational Science and Applied Research Institute, Shenzhen, 518110 China; 3 Beijing Computational Science Research Center, Beijing, 100193 China; 4 School of Information Science and Engineering, Shandong University, Shandong, 266237 China; 5 School of Information and Electronics, MIIT Key Laboratory for Low-Dimensional Quantum Structure and Devices, Beijing Institute of Technology, Beijing, 100081 China

**Keywords:** Electronic properties and materials, Molecular electronics, Electronic devices

## Abstract

Organic conjugated polymers demonstrate great potential in transistors, solar cells and light-emitting diodes, whose performances are fundamentally governed by charge transport. However, the morphology–property relationships and the underpinning charge transport mechanisms remain unclear. Particularly, whether the nonlinear charge transport in conducting polymers is appropriately formulated within non-Fermi liquids is not clear. In this work, via varying crystalline degrees of samples, we carry out systematic investigations on the charge transport nonlinearity in conducting polymers. Possible charge carriers’ dimensionality is discussed when varying the molecular chain’s crystalline orders. A heterogeneous-resistive-network (HRN) model is proposed based on the tied-link between Fermi liquids (FL) and Luttinger liquids (LL), related to the high-ordered crystalline zones and weak-coupled amorphous regions, respectively. The HRN model is supported by precise electrical and microstructural characterizations, together with theoretic evaluations, which well describes the nonlinear transport behaviors and provides new insights into the microstructure-correlated charge transport in organic solids.

## Abstract (web summary)

The mechanism of nonlinear charge transport in doped conducting polymers remains elusive. Here, Wang et al. study charge transport with respect to crystalline degrees of samples and construct a model based on the tied link between Fermi liquids and Luttinger liquids, providing a universal explanation to understand nonlinear charge transport in conducting polymers.

## Introduction

Organic conjugated polymers gained much attention for applications in flexible and logic devices due to their potential low-cost manufacturing^1,2^, and their unique carriers’ transport properties owning to the weak van der Waals interaction^3,4^. In the π-conjugated electronic systems, electrons’ delocalization robustly depends on the molecules’ stacking orders, the uncertain of which introduce considerable freedoms in micromorphology related electronic quantum states^5^. Specially, the intermediate coexisting ordered and disordered molecular arrangements makes quantitative correlation between their microstructures and the unique electrical behaviors very difficult. Several fundamental aspects about modeling the electrical transport are still not fully understood, this hinders further optimization of the materials.

In recent years, one of the most discussed electrical behaviors regarding polymers is whether their nonlinear transport behavior, i.e., the current *I* exhibits power-law dependence on both temperature *T* (*I* ∝ *T*^*α*^) and voltage *V* (*I* ∝ *V*^*β*^)^6–14^, is possibly related to Luttinger liquid (LL) features. This discussion was, to a great extent, provoked by the apparent agreements between LL theory and the experimental results for field-effect doped conducting polymers^15^. However, LL theory usually relates to strict one-dimensional (1D) systems with strong electron correlation, and its nonlinear current–voltage (*I–V*) behaviors usually stem from the power-law type density of states (DOS) near the Fermi level. Several pure 1D systems, including metallic carbon nanotubes^16–18^, nanowires^19^, and edge states in 2D systems, have been reported as LLs and show good agreement with theory^20,21^. Although isolated polymer chains have quasi-1D structures, their films are usually in condensed 2D or 3D forms, and these undoubtedly raised questions on whether the conducting polymer could be treated as collections of pure 1D LL systems without considering their detailed condensed forms on the molecular scale.

More remarkably, conducting polymers usually have complex morphologies with partially crystalline grains embedded in amorphous phase regions^22,23^. Generally, crystalline grains are formed by alignment of parallel chains with well-ordered pi–pi coupling, while the amorphous regions contain disordered chains poorly coupled with each other^24–26^. The coupling strength between the quasi-1D chains might finally determine the dimensionality of the carriers by introducing interchain’ charge delocalization. In previous works, 2D or 3D electron behaviors have been widely observed in semicrystalline doped polymers based on the Hall effect, weak localization effect^27–29^, etc., provided by crystalline grains. Interesting, the existence of 1D transport has also been suggested more than once^30,31^, especially with the decrease of interchain coherence, the quasi-1D transport is enhanced^32^. Very interesting, the studies on collections of 1D systems, such as CNT bulk samples, has already shown the inter-LL coupling strength-controlled crossover from LLs to 3D Fermi liquids (FL) under static pressure^33^, and interchain coupling effect on the electron’s dimensionality has also been discussed in polymer system recently^34^. Therefore, to unveil the origin of power-law nonlinear transport behavior in conducting polymers, the film microstructures, at least those that influence the dimensionality of carriers, should be taken into consideration.

In this work, we investigated the microstructure-dependent nonlinear transport in conducting polymers by systematically varying the crystalline degree of the samples, in which process, possible variables like charge dimensionality were introduced due to the different ratios between crystalline and amorphous regions. Nonlinear transport behavior are observed for any microstructure polymers; however, the pure LL model, together with other existed model fails to describe it. In what follows, a new charge transport mechanism is presented. By treating strongly-coupled chains in crystalline grains and weakly-coupled ones in the amorphous region as FLs and LLs, respectively, we find that the tied link between FLs and LLs accounts for the nonlinear charge transport behaviors. This conclusion has been confirmed by further experiments and theoretical evaluations demonstrating its universality. We believe the presented work can provide new insight into the charge transport behaviors in organic semiconductors.

## Results

### Crystalline-degree-dependent nonlinear transport

**Fig. 1:**
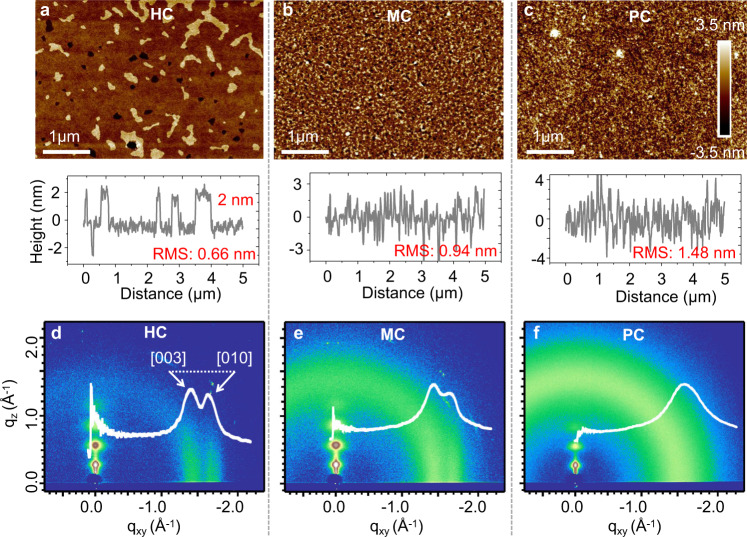
Crystalline morphology of PBTTT films. AFM characterization of polymer samples with different crystalline degrees. Surface morphology of highly crystalline (HC), moderately crystalline (MC) and poorly crystalline (PC) samples in **a**, **b**, and **c**, respectively. The surface section is displayed, with root mean square (RMS) values of surface roughness. The scale bar is 1 µm. GIWAXS for samples of different crystalline degrees, 2D diffraction images for HC (**d**), MC (**e**), and PC (**f**) samples, with the in-plane diffraction data displayed by the white curves, the peaks [003] and [010] are marked.

Semiconducting polymer films based on poly(2,5-bis(3-alkylthiophen-2-yl) thieno- [3,2-b] thiophene (PBTTT-C14, with formula shown in Supplementary Fig. S1) were prepared. The PBTTT-C14 films have different degrees of crystallinity by controlling the coating conditions. The morphology of the films was characterized by atomic force microscopy (AFM), as shown in Fig. 1a,b, c. The AFM images demonstrate apparent variations in surface roughness, revealed by the root mean square values, denoting differences in the interlayer orders of the films. Crystalline degrees were further verified by using grazing-incidence wide-angle X-ray scattering (GIWAXS), as shown in Fig. 1d,e,f. The sample shown in Fig. 1d features a so-called edge-on molecular stack, while the samples shown in Fig. 1e,f exhibit an increase in the portion of face-on diffractions. We could identify the gradual decrease in the degree of interchain pi–pi crystallinity from the quenching [010] peak. Accordingly, three samples can be noted as highly crystalline (HC) film, moderately crystalline (MC) film, and poorly crystalline (PC) film. In particular, the interchain packing-order corresponds to the electronic delocalization degree among the 1D chains and might determine the final dimensionality of charge carriers. GIWAXS spectra of the HC and PC films doped by F_4_TCNQ (2,3,5,6-Tetrafluoro-7,7,8,8- tetracyanoquinodimethane) molecules are displayed in Supplementary Fig. S1, further demonstrating the difference in packing-order or crystallinity degrees in the films.

**Fig. 2:**
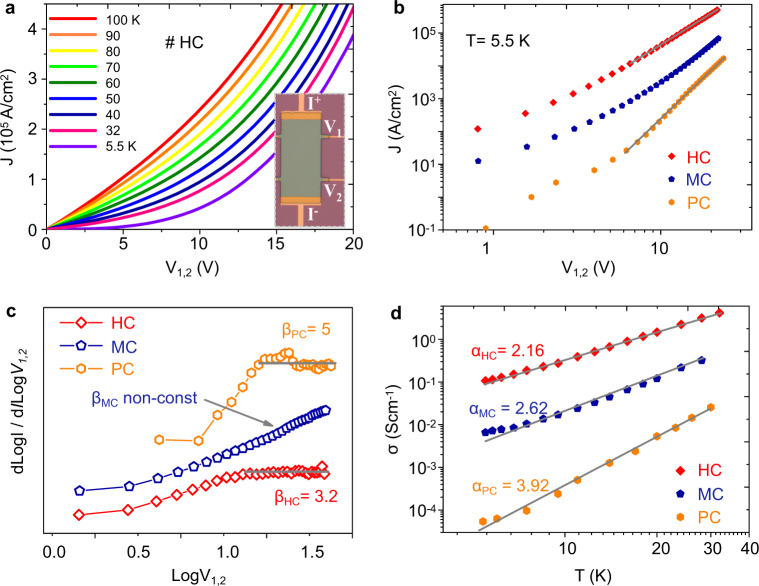
Crystalline degree-dependent nonlinear transport behaviors. **a** Nonlinear *I–V* curves obtained in a HC sample at temperatures ranging from 5.5 to 100 K. The inset shows the photo of the measured device, *I*+ and *I*− denote the current, and *V*_1,2_ is obtained by the two voltage probes, *V*_1,2_ = *V*_1_ − *V*_2_. **b** Double logarithmic-scaled *I–V* curves for HC (red), MC (blue), and PC (orange) samples at *T* = 5.5 K; the gray line denotes the power-law *I–V* relation at high voltage in HC and PC samples. **c** Plot of the values of dLog*I*/dLog*V*_1,2_ with respect to Log *V*_1,2_. The constant values of power-law exponents *β* are obtained in the HC and PC samples (*β*_PC_ = 5, *β*_HC_ = 3.2) but not in the MC sample. **d** Double logarithmic-scaled conductivity-temperature (*G–T)* relations for HC (red), MC (blue), and PC (orange) samples at temperatures ranging from *T* = 5.5–32 K. The gray line is power-law fitting of the data, with the extracted exponents α for three samples. Values of *α* (2.16, 2.62, and 3.92) were extracted within the conductivity-temperature (*G* = *dI/dV*∝*T*^*α*^) relationship.

To investigate the electrical properties of the films, we measured the devices with the four-probe configuration as displayed in the inset of Fig. 2a. Apparently, nonlinear *I–V* relationships were observed in different samples (also see Supplementary Fig. S2). Figure 2a displays the *I–V* property measured in an HC sample at low sample temperature below *T* = 100 K. After careful evaluations, we determined that the nonlinearity in the *I–V* curves (defined by curvature, *d*^2^*I/dV*^2^, Supplementary Fig. S2) was strongly dependent on the degree of crystallinity, and the power-law type relation defined by *I*∝*V*^*β*^ described the nonlinear curves of the HC and PC samples well but failed to describe the moderate MC samples.

In more detail, the curves obtained at T = 5.5 K are displayed in Fig. 2b–d with vary bias from *V*_1,2_ = 10 to 20 V. the HC and PC samples show typical power-law *I–V* relations denoted by the straight gray lines, while the MC sample displays non-power-law relations over similar bias ranges. This could be certified with the derivatives of Log*I* with respect to Log*V*_1,2,_ as shown in Fig. 2c, where constant *β* values could be obtained with *β*_*HC*_ ≈ 3.2 and *β*_*PC*_ ≈ 5, obeying the *β* = *α* + 1 relationship, while values of *α* were extracted within the conductivity–temperature (*G–T, G* = *dI/dV∝T*^*α*^) relationship (Fig. 2d). However, for MC samples, the derivative in Fig. 2c keeps increasing with no saturation, indicating a failure in the power-law description. The nonlinearity deviations for various samples mainly stem from the microstructure differences as the doping levels vary slightly among samples, as shown by the UV–visible absorption spectrum in Supplementary Fig. S3. Therefore, the pure LL theory fails in our results, it could neither cover the variations in the tunneling exponents for samples HC and PC, nor explain the non-power-law behavior in MC samples.

While LL theory fails to explain the observed crystallinity degree-dependent nonlinear transport, we turned to existing models, including nuclear tunneling, coulomb blockade, variable range hopping (VRH), etc. Although these models could to some degree describe the nonlinear *I–V* curves well, some details in extracted parameters do not obey what they predict. For examples, VRH theory claims a temperature dependent tunneling exponent, but we get a series of almost invariable *β* value at different temperatures. Detailed discussions can be found in the Supplementary Fig. S4.

### Luttinger liquid and Fermi liquids (FL–LL) networks

To unveil the origin of the invalidity of pure LL theory and microstructure-related nonlinear transport behaviors in conducting polymers, it is important to address and clarify whether/how nonlinear transport is related to the 1D nature of the polymer’s molecular structure. When dealing with this issue in conducting polymers with a 1D LL scenario, one cannot simply assume that every polymer chain is an isolated 1D electron system because 2D or 3D free electrons could exist in the highly-ordered crystalline grains^27,28^.

**Fig. 3:**
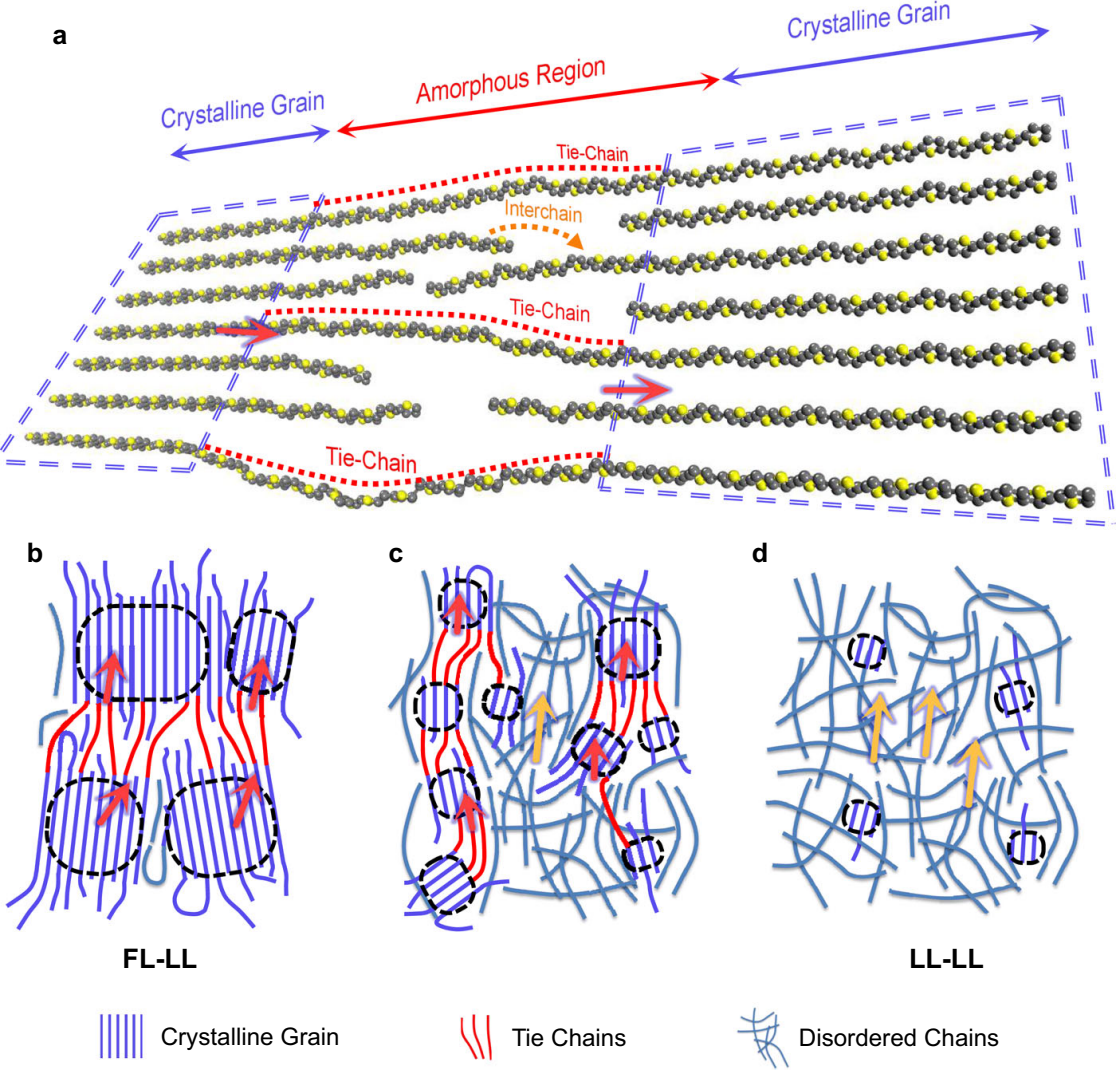
Relationship between charge transport and microstructure in polymer films. **a** Schematic illustration of the microstructure in the PBTTT film with different degrees of crystallinity, including crystalline grains with well-ordered chains, tie-chains that connect two grains, and disordered chains in the amorphous region. Schematic diagram of grain and amorphous chain distributions in PBTTT films with different degrees of crystallinity related to various possible transport paths: crystalline grain-tie-chain tunneling (denoted as red arrows) noted as FL–LL tunneling, related to the power-law in HC samples in **b**, coexisting of grain-tie-chain tunneling and chain-chain tunneling (denoted as orange arrows) in MC sample (**c**), and chain-chain tunneling in disordered PC samples, noted as LL–LL tunneling (**d**). The different dominated transport paths determine the final nonlinear transport parameters displayed in Fig. 2.

Here, we propose that a conducting polymer film is inextricably linked to the crystalline-amorphous heterogeneous network^22,25,35,36^, based on its universal semicrystalline characters, as shown in Fig. 3a. Supported by our Hall effect and 2D weak localization effect measurements shown in Supplementary Fig. S5, the well-ordered crystalline grains are believed to be metallic regions with coherent interchain transport due to the well-ordered pi–pi packing^27,37,38^, described as FL at low temperature, while the amorphous regions are dominated by weakly (incoherent) coupled chains, some of which form new percolation paths via quasi-1D conductivity through chains connecting two grains, acting as “tie-chains”^35,39–42^, marked with red dotted-lines in Fig. 3a. Based on our theoretical evaluations of the LL characteristics in quasi-1D molecules (Methods section and Supplementary Fig. S6), we treat weakly coupled chains (including tie-chains) as 1D FLs, i.e., LLs. Therefore, the conducting polymer film becomes a heterogeneous-resistive network (HRN) of FLs and LLs.

Under the above-mentioned HRN framework, the power-law *I–V* behavior could originate from one of the following scenarios: (1) FL–LL: tunneling between FLs and LLs, i.e., between crystalline grains and weakly coupled chains, as denoted by the red arrows in Fig. 3a, with power-law nonlinear *I–V* relations analyzed in Supplementary Fig. S7; (2) LL–LL: tunneling from one LL to another LL, i.., tunneling among crossed chains in the amorphous region, as marked by the orange dotted-arrow in Fig. 3a. Consequently, the collection of FLs and LLs is applied to samples with different degrees of crystallinity, as schematically shown in Fig. 3b–d. For the HC samples, transport usually occurs at paths through grains and tie-chains. Therefore, the power-law behavior originates from the tunneling junctions between FLs and LLs (Fig. 3b). For MC samples, as the grain size decreases, the increase in portions of amorphous regions leads to the coexistence of both FL–LL and LL–LL tunneling (Fig. 3c). Finally, for the PC samples, nonlinear transport mainly stems from the interchain charge transfer, i.e., tunneling between LLs (Fig. 3d).

### LL single curve scalings and transport parameters

In the HRN model, there exist both FL–LL and LL–LL types of tunneling, whose differences are determined by DOSs on either side of the tunneling junctions. Both theory and experiment show that tunneling from one LL to another LL would be more suppressed by the power-law DOS^16,17,19,43^, compared with that of FL–LL tunneling. Moreover, the power-law exponent in the *G–T* relations for the two tunnelings could be expressed as *α*_*FL–LL*_ = *λ*_*LL*_ and *α*_*LL–LL*_ = 2*λ*_*LL*_, where *λ*_LL_ are the power-law exponents for the DOS of a LL. Obviously, we have the relation *α*_*LL–LL*_ = 2*α*_*FL–LL*_.

To correlate the HRN model with the observed power-law nonlinearity behaviors, we performed fittings between *I*(*V, T*) and the dissipative tunneling equation that has always been employed to depict the tunneling current into LLs

1 $$I = I_0T^{1 + \alpha }\sinh \left( {\frac{{\gamma eV}}{{k_BT}}} \right) \cdot \left| {\Gamma \left( {\frac{{\left( {\beta + 1} \right)}}{2} + \frac{{i\gamma eV}}{{\pi k_BT}}} \right)} \right|^2.$$

where *I* is the current intensity, *I*_0_ is a prefactor, *k*_B_ is Boltzmann constant, *T* is the temperature, *e* is the elementary charge, and the reciprocal of *γ* represents the number of tunneling junctions. In a pure LL-based device, *γ* usually equals 0.5 because there are two junctions between two metal electrodes and the LL, which is not the case in our samples of the resistive network.

**Fig. 4:**
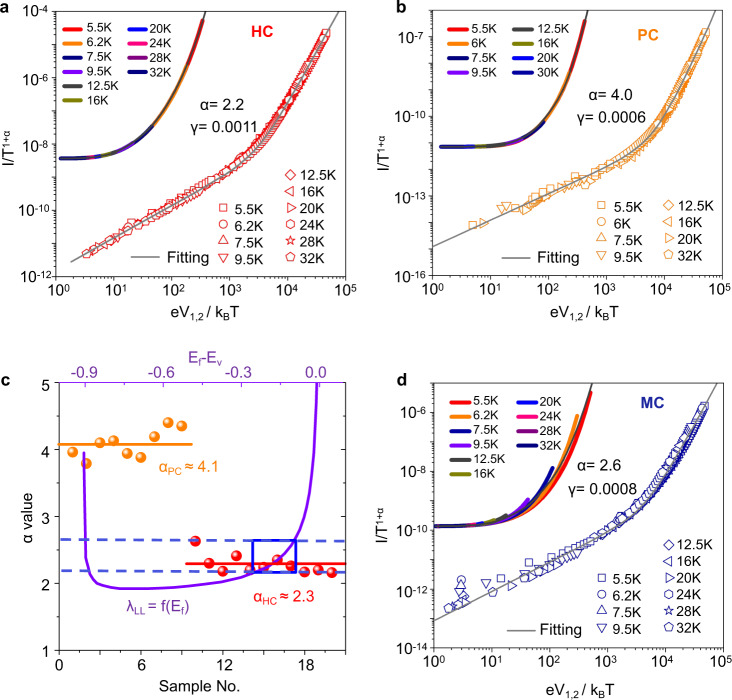
Correlation of transport parameters. Fitting between *I(V, T)* and the curves depicted by Eq. (1), for HC sample (**a**) and PC sample (**b**), which are displayed in double logarithmic coordinates. Parameters *α*, and *γ* are obtained via the fittings, and the upper-left part in the panel displays the *I(V, T)* curves in linear coordinates. **c** The distribution of the exponent α values extracted from several nonlinear curves from HC (red) and PC (orange) samples. The violet curve denotes the theoretic evaluated power-law exponent *λ*_LL_ for quasi-1D polymer chains, which is dependent on the position of the Fermi level, and equals to the experimental extracted *α*_*HC*_*’* value. The system carrier density is evaluated with the values of *α*_HC_ to *λ*_LL_ as the blue orthogon noted. **d** Fitting between *I (V, T)* and the curves depicted by Eq. (1) for the MC sample. The experimental data are more clearly identified within the linear coordinates as shown in the upper-left part in the panel.

As shown in Fig. 4a and its inset, for an HC film, multiple FL–LL tunneling junctions at tie-chain regions account for the macroscopic LL behavior well scaled with Eq. (1), with exponents of *α*_HC_ = 2.2 and *β*_HC_ = 3.2. The extracted value *γ*_HC_ = 0.0011, suggesting ~900 tunneling junctions, corresponds to an average size of 40–50 nm for one FL–LL unit, which would be further discussed with the following microstructure characterization results. When shortening the channels to lengths comparable to the grains’ sizes, as shown in Supplementary Fig S9, the nonlinearity of the *I–V* curves gradually vanished, with exponents *β*_HC_ decreasing from over 3 to ~1.5. This could be attributed to the Ohmic conductivity in tunneling between FLs (electrode-grain-electrode paths will form, when channel length is shortened to grains size) and suggests that the nonlinearity stems from the tunneling junctions containing quasi-1D weakly coupled chains.

For the PC sample shown in Fig. 4b and its inset, the collection of LL–LL tunneling junctions accounts for the well-scaled *I*(*V, T*) data, with the fitting parameters *α*_PC_ = 4, *β*_PC_ = 5 and, *γ*_PC_ = 0.0006 giving the characteristic length of *L*_LL_ ≈ 12 nm corresponding to the average intrachain transport distance along a weakly coupled chain between two interchain tunneling.

As displayed in Fig. 4c, extracted from several device features, we obtain 2.3 and 4.1 for exponents *α*_HC_ and *α*_PC_, respectively, evidently indicating the underlying relationship *α*_LL–LL_ = 2*α*_FL–LL_ between FL–LL tunneling and LL–LL tunneling, which is well consistent with the results from the mesoscopic model. Specifically, the values of exponent *α*_HC_ relating to FL–LL tunneling exponent *α*_FL–LL_, ranges from 2.2 to 2.6 and should equal to the power-law DOS exponent *λ*_LL_ in the 1D polymer chain. Combining these values with the theoretical evaluations of *λ*_LL_ shown by the purple curve (more details is available in “Methods” section and Supplementary Fig. S6), we could obtain carrier concentrations ranging from *n* = 2.5 × 10^20^ to 5.6 × 10^20^ cm^−3^ when constrain the values of *λ*_LL_ within the range of *α*_HC,_ indicated by the bule dashed-line and box, corresponding to the carrier density evaluated from the Hall effect shown in Supplementary Fig. S5, and also corresponding to the reported values in the same system characterized by electron spin resonance^29^. We further investigated the carrier density dependent nonlinear transport behavior in HC and PC samples, the evolution trend of *n* and *α* obey well with the theoretic relation of *α*_LL–LL_ = 2*α*_FL–LL_, as shown in Supplementary Fig. S10.

For the MC film, as shown in Fig. 4d, the non-power-law behavior of the *I–V* curves was not well scaled with Eq. (1), more obviously identified at linear coordinates shown in the inset. This should correspond to the coexistence of FL–LL and LL–LL tunneling. The transport would prefer FL–LL tunneling because the paths containing crystalline grains have higher conductivity, while the paths of LL–LL tunneling would become crucial at a large voltage because its resistance decreases more rapidly with voltage than that of FL-LL tunneling (*α*_LL–LL_ > *α*_FL–LL_), which leads to an increase in the *β*_MC_ value. Therefore, the non-power-law behavior stems from the voltage-induced variations in conduction paths contributed by FL–LL and LL–LL tunneling, which is a necessary phenomenon drawn by the HRN framework.

### Film microstructure: TEM characterizations

**Fig. 5:**
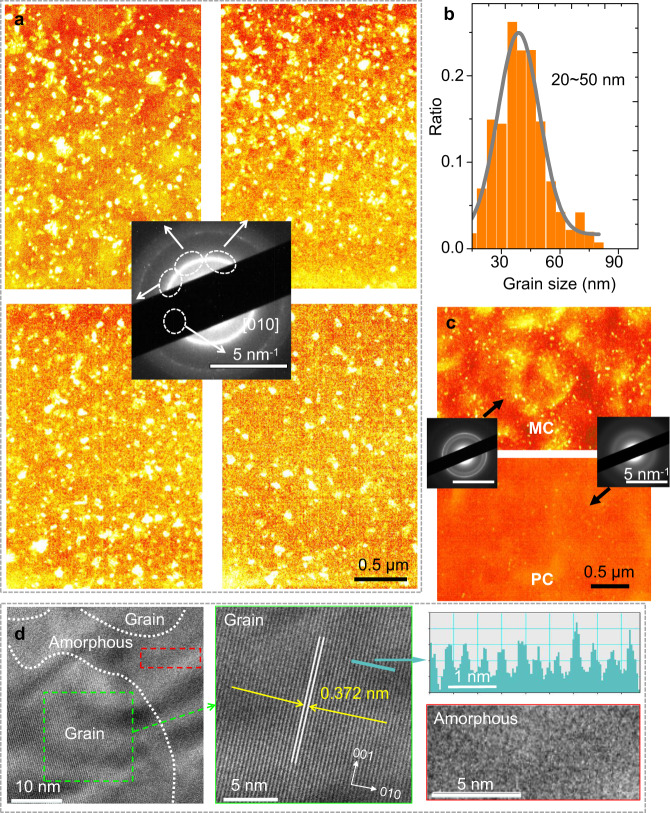
TEM and HR-TEM characterizations of the PBTTT films. **a** DF-TEM images of HC samples with four different electron-beam tilt orientations determined by the [010] face diffraction in SAED shown in the inset. **b** Grain size distribution in the HC sample. The distribution could be fitted with a Gaussian distribution function centered at ~35 nm. **c** DF-TEM image of MC and PC samples featuring fewer and smaller grains; the inset shows the SAED of MC and PC samples. The scale bars for the DF-TEM images are 0.5 µm, and 5 nm^−1^ for the SAED images. **d** HR-TEM characterization of HC samples at different zoom scales and regions, with scale bar *L* = 10, 5, 5 nm for the left, middle, and right (down), respectively. The middle and right (down) display the detailed morphology for crystalline grain and amorphous region marked by green and red box in the left panel, respectively. The [010] pi–pi face is identified with lattice constant *a* = 0.372 nm, more precisely shown in upper-right, the intensity cross profile of the inter chain lattice is displayed with scale bar *L* = 1 nm.

We have qualitatively classified the samples into HC, MC, and PC ones as above, further quantification is needed to analyze the microstructure characters of the polymer films to the nonlinear electrical behavior within the HRN frameworks, specifically, mesoscopic parameters like crystalline grains’ spatial distribution should be clarified. Here, dark-field transmission electron microscopy (DF-TEM) was employed to unveil the crystalline details of the films. Selective area electron diffraction provided an in-plane diffraction patterns of different samples. The DF-TEM characterizations were realized by locking on one specific arc of the [010] ring while tilting the incident electron beam. Thus, only grains with lattice orientations consistent with the locked diffraction arc could let most electrons pass the centered objective aperture and exhibit the brightest image. The DF-TEM images in Fig. 5a show four different tilt orientations for HC samples. Thus the HC film has isotropic crystallization deduced from the similar density of grains distribution for different lattice orientations. The character size of the grains is estimated with the statistical measurement of random grains. The grain size distribution is displayed in Fig. 5b. The average size is *L*_G_ ≈ 35 nm, much smaller than the length of the polymer chains (*L*_chain_ ≈ 100 nm) in our sample, which is a necessary condition for the formation of tie-chains. Crystalline grains could also be identified from the transmission electron beam contrast with bare copper grids, with an average grain size of approximately *L*_G_ ≈ 40 nm, as shown in Supplementary Fig. S8. The crystalline grain size distribution is well consistent with the extracted results from the electrical transport analyzed in the HRN scenario, which gives an average spatial extension of 40–50 nm for a FL–LL tunneling unit. For comparison, the DF-TEM images of both MC and PC samples are shown in Fig. 5c. The grains are distributed more sparsely and the size is smaller for MC sample, while the PC sample have less grains. More details about comparison among three samples are displayed in Supplementary Table S1.

High-resolution transmission electron microscopy characterizations were performed on PBTTT films of HC samples to gain more insight into the detailed structures. As shown in Fig. 5d, the regions of evident [010] stripe with an interval distance of *a* = 0.372 nm, which coincide well with the reported lattice constant^26^, could be identified as highly crystalline grains. The intergrain amorphous region exhibits a spatial extension of ~10–20 nm, suggesting that the average size of a grain/intergrain unit in HC samples is ~30–60 nm, which confirms the FL-LL unit in the assumed HRN framework, and the TEM characterizations support well three HRN networks demonstrated in Fig. 3 and Supplementary Fig. S7.

## Discussion

Heterogeneous analysis in dealing with disordered condensed materials, such as the grain boundary (GB) model in inorganic polycrystalline silicons, has proven to be an efficient approach^44^. While differing from inorganic or small-molecule materials, crystalline polymers have intergrain regions with much larger spatial extensions of up to tens of nanometers. Our HRN model emphasized that charge carriers’ intergrain transport is mainly realized by quasi-1D intrachain transport through the tie-chain structure, which is necessary for LL’ dominant behaviors in low-*T* nonlinear transport. One may argue that the intergrain incoherent hopping could also contribute to the GBs’ conductance. However, we found that a zero-voltage conductivity up to 0.1–5 S cm^−1^ was obtained at temperatures ranging from 5.5 to 32 K, as shown in Fig. 2d. This corresponds to a mobility of approximately 10^−3^–10^−1^ cm^2^ V^−1^ s^−1^, which can hardly be expected in long-range hopping over GBs in such low T without any other activation factors (electric field **E** ≈ 0). In addition, intergrain transition have usually been related to fluctuation induced tunneling (FIT) transport, which is hardly applicative to our data shown in Supplementary Fig. S4e. Another concern is whether crystalline grains are 2D LLs, such as the case in organic bulk conductor TTF:TCNQ^45^. This possibility was discounted by the electrical characterizations on submicrometer channel devices, as shown in Supplementary Fig. S9. When the narrowed conducting channels were partially dominated by single grains, the nonlinearity in *I–V* curves diminished, indicating the ohmic transport behavior as described in conventional FLs.

**Fig. 6:**
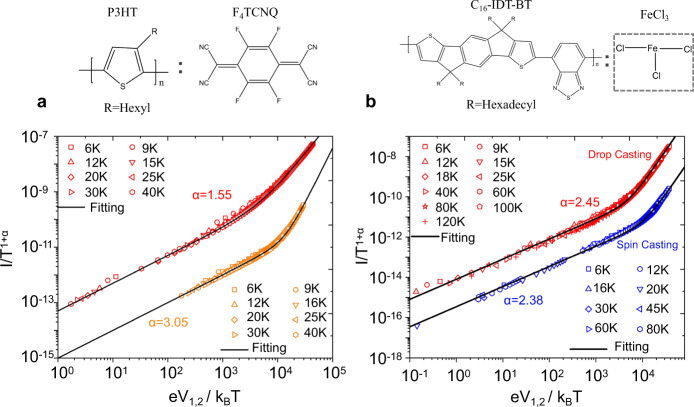
Scaling of nonlinear transport behavior of doped conducting polymer samples based on P3HT and IDT-BT. **a** Crystalline polymer of Poly(3-hexylthiophene) (P3HT) doped with F_4_TCNQ. Fitting between *I(V, T)* and the curves depicted by Eq. (1), for HC P3HT sample (red) and PC one (orange), which are displayed in double logarithmic coordinates. Structural formulas of P3HT and dopant F_4_TCNQ are shown at top. **b** Noncrystalline polymer of indacenodithiophene-benzothiadiazole (IDT-BT) doped with FeCl_3_. Fitting between *I(V, T)* and the curves depicted by Eq. (1), for drop casting IDT-BT sample (red) and spin casting one (blue), which are displayed in double logarithmic coordinates. Structural formulas of IDT-BT and dopant FeCl_3_ are shown at top. Values of parameters *α* are obtained via the fittings.

Aiming to apply the proposed hybrid FL–LL network to a universal model, we further characterized the low-T transport for other crystalline and noncrystalline polymers. Typically, crystalline film of Poly(3-hexylthiophene) (P3HT) doped with F_4_TCNQ and noncrystalline film of indacenodithiophene-benzothiadiazole (IDT-BT)^46^ doped with FeCl_3_. The conducting P3HT film also showed power-law nonlinearity in *I–V* relations at highly crystalline and amorphous limits, respectively, which could universally be scaled with Eq. (1), as shown in Fig. 6a. The extracted power-law exponents are *α*_HC_ = 1.55 and *α*_PC_ = 3.05, fulfilling the relationship *α*_PC_ ≈ 2*α*_HC_ predicted in our theoretic frameworks. While for doped IDT-BT film, no matter what film process conditions were employed, samples show similar values of power-law exponents when fitting the *I–V* data by the universal scaling curves displayed in Fig. 6b and in Supplementary Table SI. This phenomenon could also be included in our model, as IDT-BT is a less crystalline material, 2D FL regions are absent in the framework, where charge transport only occurs via LL–LL tunnelings, the power-law exponent take on a single value *α* = *α*_LL–LL_. More details for various samples of P3HT and IDT-BT can be found in Supplementary Table S2.

We investigated the nonlinear transport behaviors in conducting polymers by considering the influence of microstructures at the molecular level by varying their degree of crystallinity. We proposed a mesoscopic model in which the conducting polymers are systems of HRN by tied link from the Fermi liquids and Luttinger liquids, corresponding to a tied hybrid framework of crystalline grains and noncrystalline weakly-coupled chains, respectively. The nonlinear transport behaviors with power-law *I–V* curves are attributed to the carrier tunneling between FL and LL in highly-ordered films or tunneling among LLs in amorphous films, which are well supported by experimental characterizations and theoretical evaluations. We believe our HRN model could open a pathway for understanding the universal nonlinear transport phenomenon in conducting polymer systems and help optimize the design of polymer materials.

## Methods

### Conducting polymer film preparation

HC polymer film: A PBTTT solution at a concentration of 5 mg/ml was prepared by dissolving PBTTT in dichlorobenzene at 150 °C. Then, the solution was spin-coated onto an OTS-treated SiO_2_ substrate, forming PBTTT layer of about 20 nm thickness. After which, the sample was annealed at 150 °C for 10 min and allowed to slowly cool. MC polymer film: Coating was performed as for the HC film. The substrate was SiO_2_ without OTS modification and without annealing. PC polymer film: A PBTTT solution at a concentration of 10 mg/ml was prepared by dissolving PBTTT in chloroform at 60 °C. Then, the solution was spin-coated onto a heated SiO_2_ substrate, the SiO_2_ substrate was pretreated with exposure to HF gas from a 20% wt HF solution for 10 s, without annealing, the films’ thickness is around 40–50 nm.

#### F_4_TCNQ doping

F_4_TCNQ molecules were vacuum-evaporated onto the PBTTT film in an AUTO 306 physical vacuum deposition system, at a deposition rate of 0.02 nm/s, with a final nominal thickness of 50 nm, the vacuum pressure was about 3 × 10^−5^ Pa.

### Material characterizations

GIWAXS: The structures were measured using a Xeuss SAXS/WAXS system with Xenocs Genix Cu ULD and Dectris 100 K Pilatus. GIWAXS measurements were performed on the 7.3.3 beamline at the Advanced Light Source (ALS). The 10 keV X-ray beam was incident at a grazing angle of 0.12°–0.15° and selected to maximize the scattering intensity from the samples. Scattered X-rays were detected by using a Dectris Pilatus 2 M photon counting detector. AFM: Surface morphology characterization was performed on a Bruker MultiMode-V AFM in tapping mode, with scanning frequency *f* = 0.3. TEM: (1) TEM specimen preparation: polymer film-coated SiO_2_/Si substrates were dipped into BOE solvent to etch the SiO_2_ layer, and then the organic films were transferred onto the water surface and picked up by a copper grid. The residual water was removed by placing the samples into a vacuum chamber overnight. (2) Characterization: A JEOL JEM-2100 TEM, operated at 200 kV, was used to perform dark field imaging and electron diffraction, and a JEOL JEM-2100F TEM, operated at 200 kV, was used to perform high resolution imaging. To suppress the degradation of the organic layer under the electron beam, a cryogenic sample holder was employed to keep the sample at low temperature *T* = 90 K.

### Devices fabrication

Electrode contacts were formed via ultraviolet lithography (UV lithography) and electron beam evaporation of 5 nm Ti followed by 25 nm Au, with an anode-cathode length of *L* = 40 µm and a distance of *l* = 20 µm between the two voltage probes. Finally, the polymer was coated onto the substrate with patterned electrodes, followed by molecular doping. Patterning of conducting polymer was realized by UV lithography and oxygen plasma etching, with a parylene layer between the conducting channel and photoresist layer for protection. Devices with submicrometer channels were fabricated with transferred and patterned graphene electrodes. The channels were formed by electron beam lithography and oxygen ion plasma etching. Voltage probes of 10 nm scale were formed by helium ion deposition of platinum to exclude graphene electrode resistance.

### Electrical measurements

Electrical characterizations were carried out by employing a Keithley 4200 semiconductor parameter analyzer together with a Lakeshore four-probe-station system or a Quantum designed physical properties measurement system. Fifteen minutes was needed for the device’s thermal equilibrium before measurement for every temperature point. The *I–V* curves were achieved by setting a voltage sweeping mode in the analyzer, where the *I*_+_ and *I*_−_ electrodes could record the current, while *V*_1_ and *V*_2_ could record the voltage drop in the channel.

### DFT calculations

Density functional theory calculations were performed by using the Vienna Ab Initio Simulation Package^47^ with the projector augmented wave basis sets^48^ and the generalized gradient approximation^49^ to the exchange correlation potential. The PBTTT-C14 polymer is modeled by employing an orthorhombic unit cell with a repeat backbone along the *b* direction and side chains along the *c* direction. In this paper, we did not consider the tilting of the side chains. The lattice constants in the *a* and *c* directions were taken to be 17 Å and 50 Å, respectively, to reduce the interaction between the successive unit cells. The length of the backbone, i.e., the lattice constant in the b direction, was set to the value (13.77 Å) with the lowest energy in the calculated energy vs. lattice constant curve. Then, the atomic positions were fully relaxed until the force on each atom was <0.01 eV/Å. The mesh of *k*-point sampling during optimizations was 1 × 3 × 1, and the plan wave cutoff energy was 400 eV. In the calculations of the band structure and density of states, we kept the cutoff energy of 400 eV but increased the total number of *k*-points to 199. The Gaussian smearing method with a broadening of 0.01 eV was employed for the integration in the Brillouin zone.

## Supplementary information

Supplementary Information

## Data Availability

All data needed to evaluate the conclusions in the paper are present in the paper and/or the Supplementary Information. Additional data related to this paper may be requested from the authors.

## References

[1] Guo X (2017). Current status and opportunities of organic thin-film transistor technologies. IEEE Trans. Electron Devices.

[2] Chang JS, Facchetti AF, Reuss R (2017). A circuits and systems perspective of organic/printed electronics: review, challenges, and contemporary and emerging design approaches. IEEE J. Em. Sel. Top. C.

[3] Coropceanu V (2007). Charge transport in organic semiconductors. Chem. Rev..

[4] Nelson J, Kwiatkowski JJ, Kirkpatrick J, Frost JM (2009). Modeling charge transport in organic photovoltaic materials. Acc. Chem. Res..

[5] Fratini S (2020). Charge transport in high-mobility conjugated polymers and molecular semiconductors. Nat. Mater..

[6] Aleshin AN, Lee HJ, Park YW, Akagi K (2004). One-dimensional transport in polymer nanofibers. Phys. Rev. Lett..

[7] Kronemeijer AJ (2010). Universal scaling in highly doped conducting polymer films. Phys. Rev. Lett..

[8] Park JG (2003). Tunneling conduction in polyacetylene nanofiber. Synth. Met..

[9] Yu D (2004). Variable range hopping conduction in semiconductor nanocrystal solids. Phys. Rev. Lett..

[10] Asadi K (2013). Polaron hopping mediated by nuclear tunnelling in semiconducting polymers at high carrier density. Nat. Comm..

[11] Li L (2014). Physical origin of nonlinear transport in organic semiconductor at high carrier densities. J. Appl. Phys..

[12] Akai-Kasaya M (2015). Coulomb blockade in a two-dimensional conductive polymer monolayer. Phys. Rev. Lett..

[13] Abdalla H (2015). Effective temperature and universal conductivity scaling in organic semiconductors. Sci. Rep..

[14] Kronemeijer A (2011). Universal scaling of the charge transport in large-area molecular junctions. Small.

[15] Yuen JD (2009). Nonlinear transport in semiconducting polymers at high carrier densities. Nat. Mater..

[16] Yao Z (1999). Carbon nanotube intramolecular junctions. Nature.

[17] Gao B (2004). Evidence for Luttinger-liquid behavior in crossed metallic single-wall nanotubes. Phys. Rev. Lett..

[18] Bockrath M (1999). Luttinger-liquid behaviour in carbon nanotubes. Nature.

[19] Venkataraman L (2006). Electron transport in a multichannel one-dimensional conductor: molybdenum selenide nanowires. Phys. Rev. Lett..

[20] Stühler R (2020). Tomonaga-Luttinger liquid in the edge channels of a quantum spin Hall insulator. Nat. Phys..

[21] Yang G (2020). Possible Luttinger liquid behavior of edge transport in monolayer transition metal dichalcogenide crystals. Nat. Comm..

[22] Noriega R (2013). A general relationship between disorder, aggregation and charge transport in conjugated polymers. Nat. Mater..

[23] Luzio A (2019). Microstructural control suppresses thermal activation of electron transport at room temperature in polymer transistors. Nat. Comm..

[24] Joo J (1998). Charge transport of the mesoscopic metallic state in partially crystalline polyanilines. Phys. Rev. B.

[25] Himmelberger S (2013). Effects of confinement on microstructure and charge transport in high performance semicrystalline polymer semiconductors. Adv. Funct. Mater..

[26] Patel S (2017). Morphology controls the thermoelectric power factor of a doped semiconducting polymer. Sci. Adv..

[27] Kang K (2016). 2D coherent charge transport in highly ordered conducting polymers doped by solid state diffusion. Nat. Mater..

[28] Fujimoto R (2017). Control of molecular doping in conjugated polymers by thermal annealing. Org. Electron..

[29] Honma Y (2018). Mesoscopic 2D charge transport in commonplace PEDOT:PSS films. Adv. Electron. Mater..

[30] van de Ruit Kevin (2013). Quasi-one dimensional in-plane conductivity in filamentary films of PEDOT:PSS. Adv. Func. Mater..

[31] Reedijk J (1999). Dopant-induced crossover from 1D to 3D charge transport in conjugated polymers. Phys. Rev. Lett..

[32] Wang Z (1990). Electron localization and charge transport in poly(o-toluidine): a model polyaniline derivative. Phys. Rev. B.

[33] Monteverde M (2006). Tomonaga-Luttinger liquid and coulomb blockade in multiwall carbon nanotubes under pressure. Phys. Rev. Lett..

[34] Szasz A (2017). Electrical and thermal transport in the quasiatomic limit of coupled Luttinger liquids. Phys. Rev. B.

[35] Mollinger SA (2015). Percolation, tie-molecules, and the microstructural determinants of charge transport in semicrystalline conjugated polymers. ACS Macro. Lett..

[36] Zhang X (2010). In-plane liquid crystalline texture of high-performance thienothiophene copolymer thin films. Adv. Funct. Mater..

[37] Kaiser AB (2001). Electronic transport properties of conducting polymers and carbon nanotubes. Rep. Prog. Phys..

[38] Miller ED (2018). Tying together multiscale calculations for charge transport in p3ht: structural descriptors, morphology, and tie-chains. Polymers.

[39] Pingel P (2010). Temperature-resolved local and macroscopic charge carrier transport in thin P3HT layers. Adv. Funct. Mater..

[40] Segatta F (2018). Predicting charge mobility of organic semiconductors with complex morphology. Macromolecules.

[41] Gu K (2018). Assessing the Huang−Brown description of tie chains for charge transport in conjugated polymers. ACS Macro. Lett..

[42] Schott S (2015). Charge-transport anisotropy in a uniaxially aligned diketopyrrolopyrrole-based copolymer. Adv. Mater..

[43] Moser J (1998). Transverse transport in (TM)2X organic conductors: possible evidence for a Luttinger liquid. Eur. Phys. J. B.

[44] Seto John (1975). The electrical properties of polycrystalline silicon films. J. Appl. Phys..

[45] Ito T (2005). Temperature-dependent luttinger surfaces. Phys. Rev. Lett..

[46] Schott S (2019). Polaron spin dynamics in high-mobility polymeric semiconductors. Nat. Phys..

[47] Kresse G (1996). Efficient iterative schemes for ab initio total-energy calculations using a plane-wave basis set. Phys. Rev. B.

[48] Blöchl PE (1994). Projector augmented-wave method. Phys. Rev. B.

[49] Perdew JP (1996). Generalized gradient approximation made simple. Phys. Rev. Lett..

