# Supplementary material for: A tied Fermi liquid to Luttinger liquid model for nonlinear transport in conducting polymers
Source: Nat Commun. 2021 Jan 4;12:58. doi: 10.1038/s41467-020-20238-5 (PMC7782818; doi:10.1038/s41467-020-20238-5)
Supplement: Supplementary file 1 — Supplementary Information [file 41467_2020_20238_MOESM1_ESM.pdf]

Supplementary Information

A tied Fermi liquid to Luttinger liquid model for nonlinear transport in conducting polymers

Jiawei Wang[1], Jiebin Niu[1], Bin Shao[2,3], Guanhua Yang[1], Congyan Lu[1], Mengmeng Li[1], Zheng Zhou[1], Xichen Chuai[1], Jiezhi Chen[4], Nianduan Lu[1], Bing Huang[3], Yeliang Wang[5*], Ling Li[1*], and Ming Liu[1*]

[1]Key Laboratory of Microelectronic Devices & Integrated Technology, Institute of Microelectronics, Chinese Academy of Sciences, Beijing 100029, China.

[2]Shenzhen JL Computational Science and Applied Research Institute, Shenzhen 518110, China

[3]Beijing Computational Science Research Center, Beijing 100193, China

[4]School of Information Science and Engineering, Shandong University, Shandong 266237, China

[5]School of Information and Electronics, MIIT Key Laboratory for Low-Dimensional Quantum Structure and Devices, Beijing Institute of Technology, Beijing 100081, China.

*E-mails: yeliang.wang@bit.edu.cn; lingli@ime.ac.cn; liuming@ime.ac.cn

Content:

Part.1 GIWAXS and ESR data of F_4TCNQ doped PBTTT film

Part.2 Device configuration and the basic electrical properties

Part.3 UV-Visible absorption spectrums for PBTTT films

Part.4 Comparison between different models in depictions of the nonlinear transport in PBTTT : F_4TCNQ

Part.5 HALL and magnetoresistance of doped PBTTT films of different crystalline degree.

Part.6 Theoretic evaluation of the power law nature in the 1D polymer chain by First principle calculations with 1D Bosonization approach

Part.7 Schematic diagram of the equivalent circuit of the Fermi liquid - Luttinger liquid resistive network

Part.8 TEM morphology of HC PBTTT film

Part.9 Submicrometer channel device for nonlinear transport investigations

Part.10 Doping degree dependent nonlinear transport in PBTTT samples

Part.11 Grain size, grain density, and diffraction ring's HWHM of three type samples

Part.12 Statistical values of conductivity σ and power-law exponents α for doped P_3HT and IDT-BT

Part.13 Supplementary Note 1

Part.14 Supplementary Note 2

Part.1

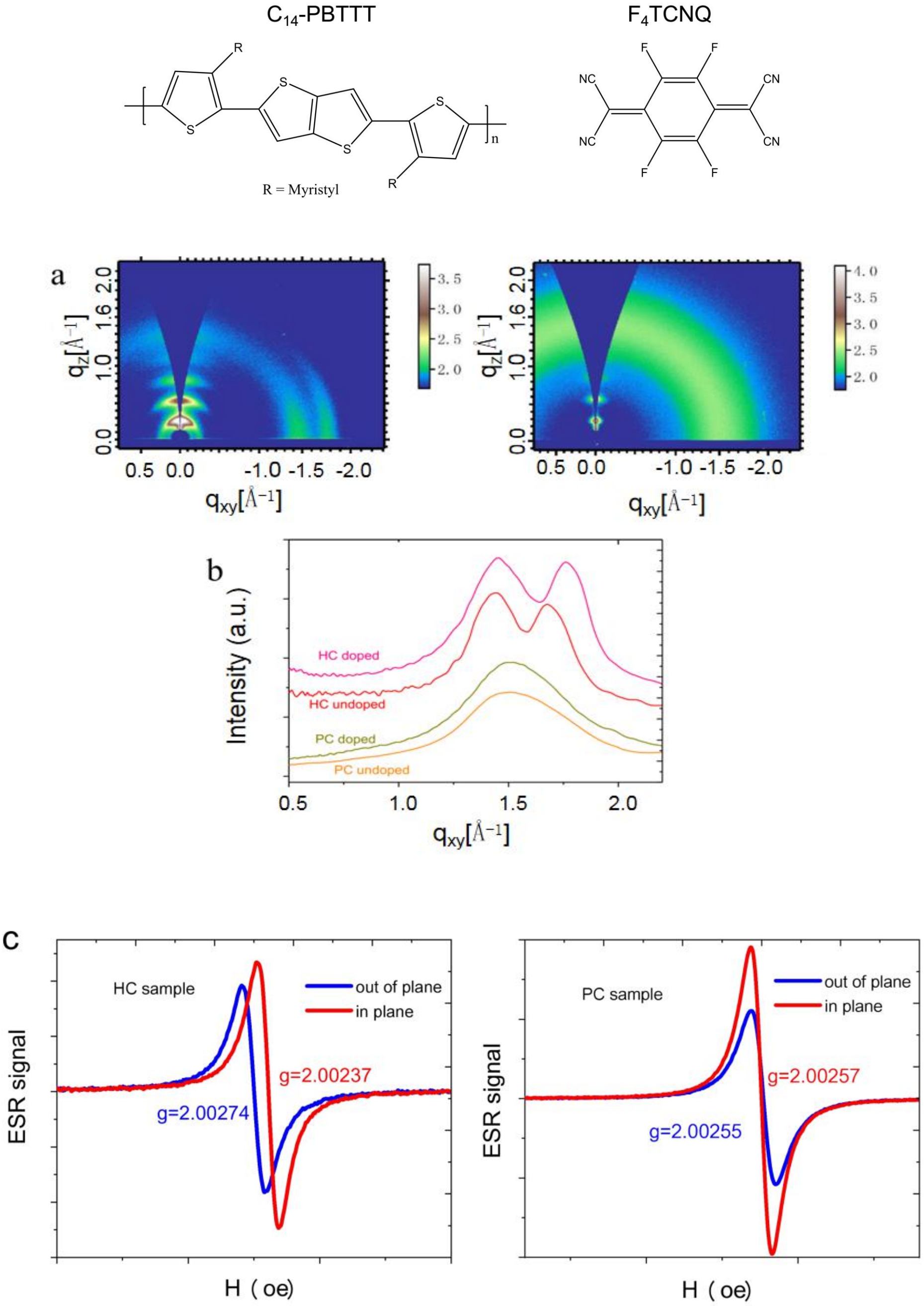

Supplementary Figure 1. The molecular structures of PBTTT-C14 and the doping molecule F_4TCNQ are shown at the top. a. GIWAXS of doped HC (left) and PC samples (right). b. Comparison between doped and pristine samples. c. Electron spin resonance (ESR) characterizations of doped HC and PC samples.

For highly crystalline (HC) samples, the doping process could enhance the pi-pi coupling along the [010] direction by suppressing the lattice constant (with the shift of the peak), however, for the poorly crystalline (PC) samples, no enhancement of the crystalline degree of the [010] pi-pi

stacking could be identified. The Lande factors extracted from ESR indicates the crystalline related anisotropic spin-orbit coupling strength in HC sample, while the PC sample shows amorphous nature with isotropic Lande factors.

Part. 2

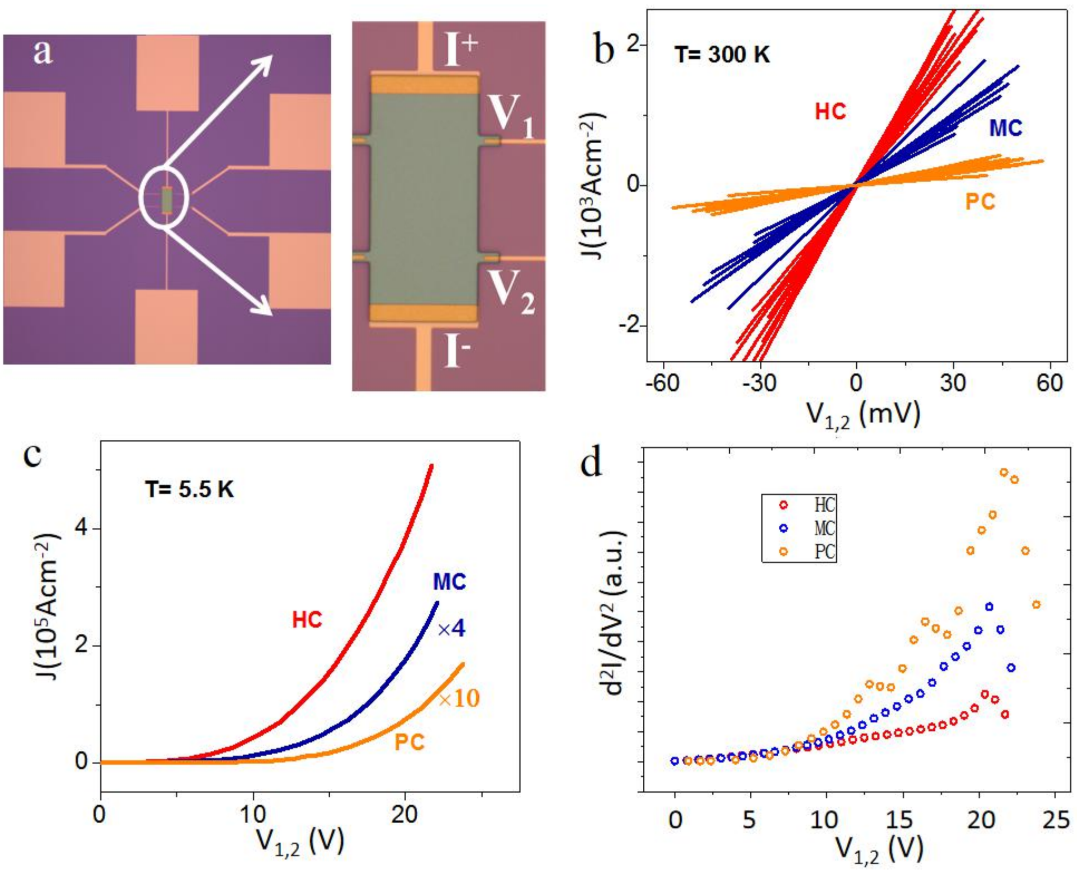

Supplementary Figure 2. Device configuration and the basic electrical properties. a. Optic images of four terminal (4T) devices for the electrical characterizations of doped PBTTT films. Device patterning was realized by UV lithography and Oxygen plasma etching, with a perylene layer protecting the doped polymer. **b**. Linear *I-V* curves extracted from tens devices of HC (red), MC (blue), and PC (orange) samples at T = 300 K. The average mobility of three types of samples are u=1.35, 0.51, and 0.12 $cm^2V^{-1}s^{-1}$ for HC, MC, and PC samples, respectively. **c.** Nonlinear *I-V* curves for devices of HC (red), MC (blue), and PC (orange) samples at T = 5.5 K. **d.** Nonlinearity of *I-V* curves for HC (red circle), MC (blue circle), and PC (orange circle) samples at T = 5.5 K.

Part. 3

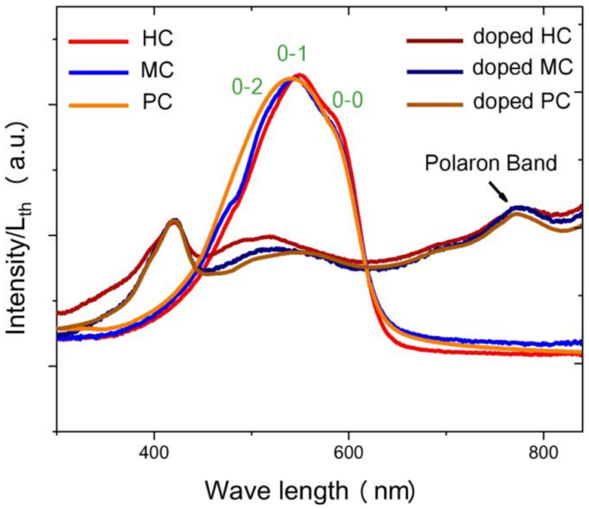

Supplementary Figure 3. UV-Visible absorption spectrums for PBTTT films of HC (red), MC (blue), and PC (orange) samples. The absorptions of pristine and doped films are displayed by circles and dots, respectively. To compare the carrier concentrations or the doping levels, the equation $\frac{\Delta T}{T} \sim L_{\mathrm{th}} \cdot n$ is employed, where ΔT is the light transmission difference at the polaron band absorption, which is could be extracted with the experiment data, L_{th} is the film thickness. The doping degrees for three types of samples are in similar level from the values of $\Delta T/L_{th}$. Besides, the difference in crystalline degrees could also be verified from the ratio of peaks of 0-0, 0-1, 0-2, and higher-level transitions. The 0-0 peak in HC sample is strongest than other samples, indicating the highest crystalline degree among the samples.

Part. 4a

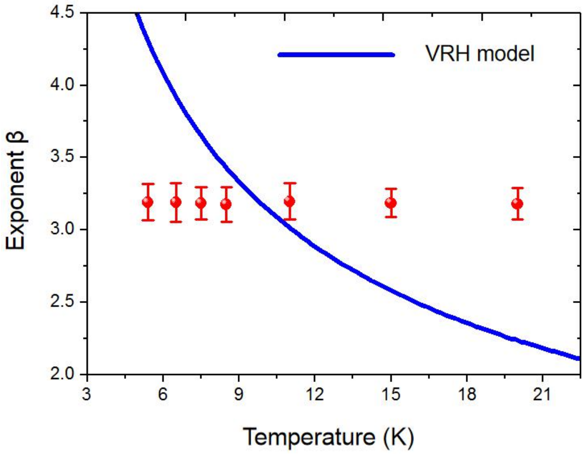

Supplementary Figure 4a. Relationship between power-law exponent β and temperature T. It's clear that the values of β (red circles) extracted in HC samples are almost constant at T ranging from 5.5 K to 20 K, deviating from the functions $\beta \sim T^{-0.5}$ (blue curve) from 1D VRH theory [1].

Part. 4b

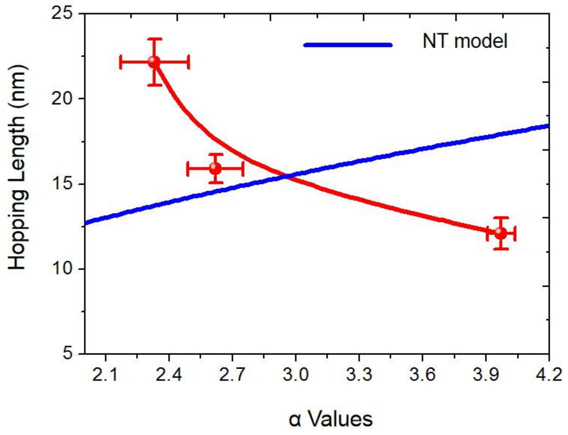

Supplementary Figure 4b. Relationship between hopping length L_{ij} and α. Developed from the double well dissipation tunneling system. Nuclear tunneling (NT) theory predicts a α-L relation$\alpha = \eta L^2/2\pi\hbar$, as depicted with the blue curve. This illustrated well the α-L_{ij} relations from our experimental data (red symbols) [2,3].

Part. 4c

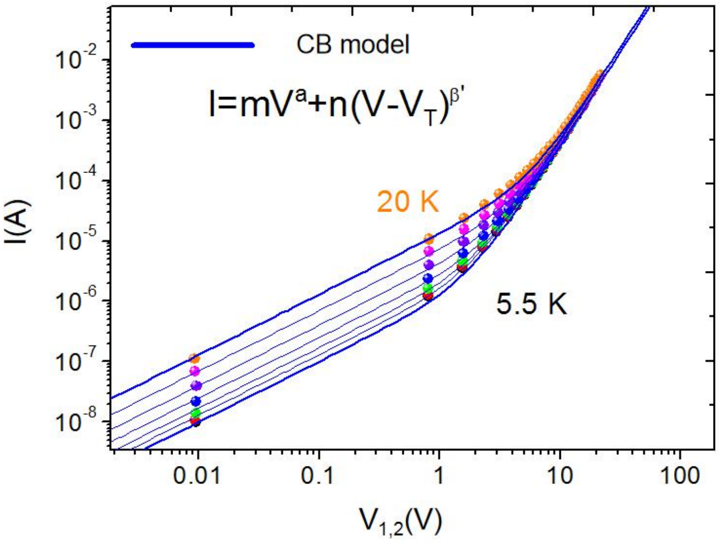

Supplementary Figure 4c. Fitting the low-T data with Coulomb blockade (CB) model. The fitting is conducted via the formula $I = mV^a + n(V - V_T)^{\beta'}$, in which, β' is independent of temperature; *m* and *n* are a constant especially at temperatures below *T**, *a* has a value of 1 above *T**. *T** is the effective temperature below which the CB effect takes place [4]. The fitting parameters are:

0.000013*x + 0.00000035*(x)^3.1

0.000007*x + 0.00000034*(x)^3.1

0.000004*x + 0.00000034*(x)^3.1

0.0000025*x + 0.00000033*(x)^3.1

0.0000017*x + 0.00000032*(x)^3.1

0.0000013*x + 0.00000031*(x)^3.1

0.000001*x + 0.0000003*(x)^3.1,

The values of V_T at all temperature are 0 V, and the values of *a* are constant as 1, *m* and *n* are variables, indicating the temperature is above *T**. No macroscopic CB effect could be observed.

Part. 4d

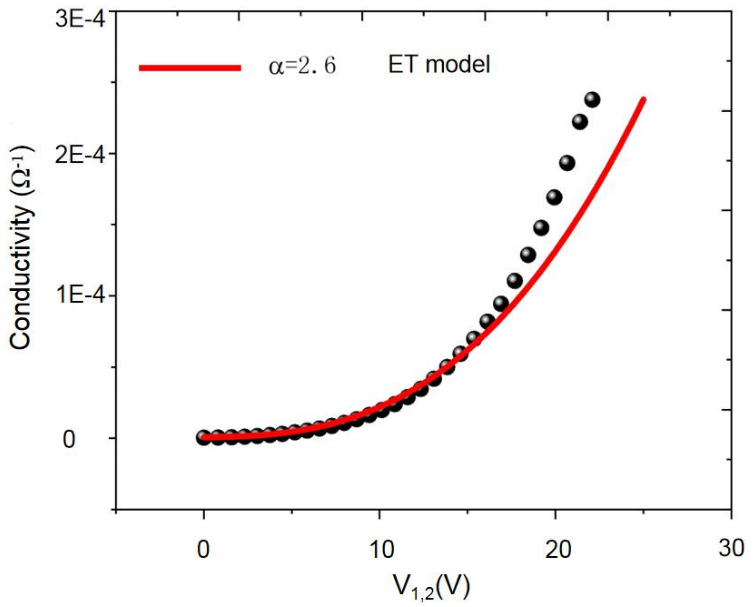

Supplementary Figure 4d. Fitting the MC samples data with effective temperature model (ET). This ET model predicts a strict power-law relationship between *I* and *V* at high bias limit as $\sigma \sim \left(\sqrt{(T_{lattice})^2 + (\frac{2}{3}\frac{eV}{k_B N})^2}\right)^{\alpha}$. The MC samples exhibited a super-power-law nonlinearity, which deviate what the ET model's prediction. Thus, the ET model could also be excluded [5].

Part. 4e

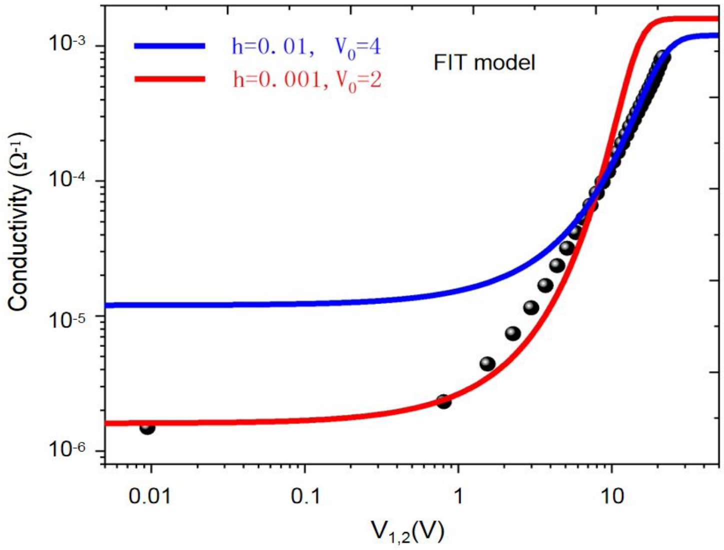

Supplementary Figure 4e. Fitting the HC sample data with fluctuation induced tunneling model (FIT). This FIT model predicts a relationship between conductivity σ and V, $\sigma = \sigma_0 \frac{exp(V/V_0)}{1+h(exp(V/V_0)-1)}$, which could not well fit the σ-V curve [6]. Physically, FIT relates to the charge hopping among segments, in each of which, carriers could be treated as delocalization to some degree, as the situations in para-crystalline materials with large domains.

Part. 4f

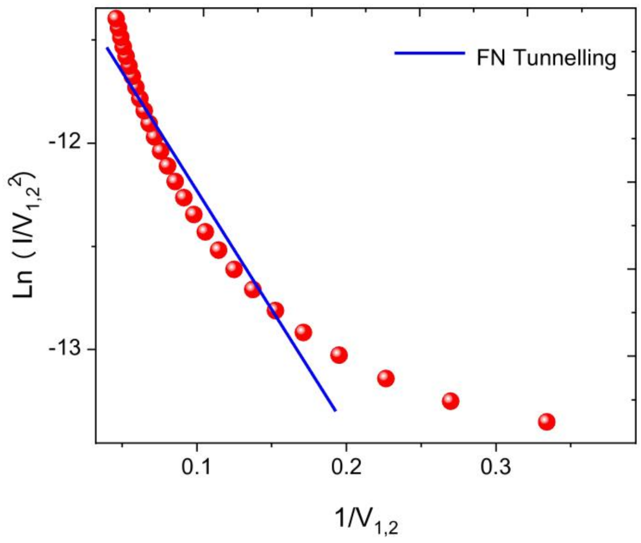

Supplementary Figure 4f. Fitting the HC PBTTT sample with the multi-barrier FN tunneling model. The FN model predicts a relationship between current *I* and voltage *V*, $I = V^2 \exp(-\frac{4d\sqrt{2m^*\phi}}{3hqV})$ [7], this model was used to fit the nonlinear transport in PBTTT transistor, whose conductivity is five orders of magnitude lower than the doped PBTTT in our work, the model could not fit our experimental data.

Part. 5a

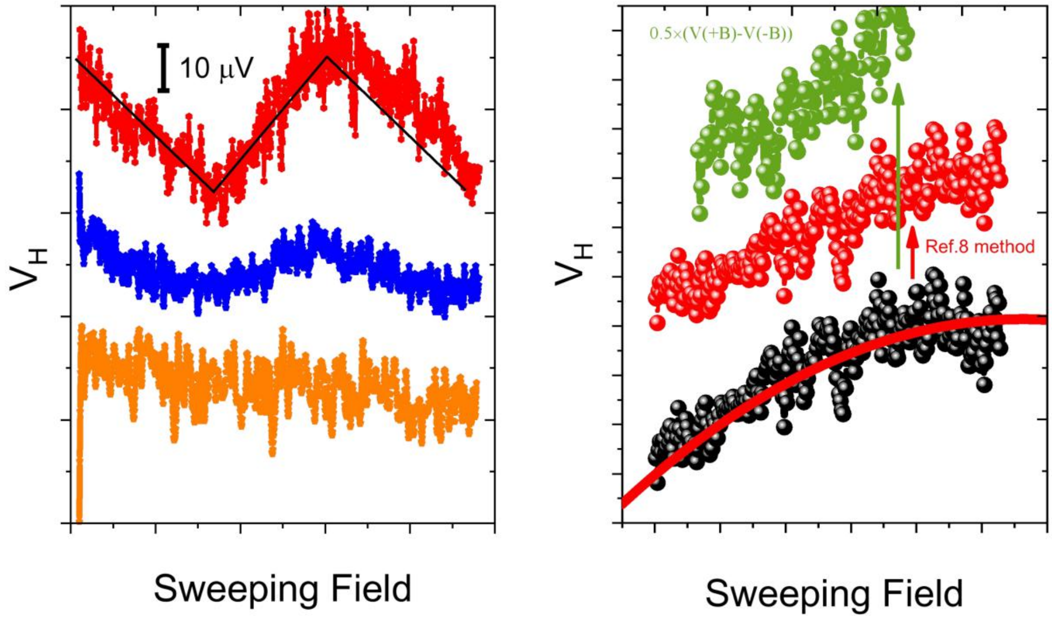

Supplementary Figure 5a. Hall effect measurements for devices of HC, MC and PC samples (left), and to subtract the voltage contributed by other factors like offset voltage and magnetoresistance voltage (right).

HC samples exhibit Hall voltage of tens nanovolts, corresponding with carrier concentration n_{hc} = 3~7*10^{20} cm^{-3}, a reasonable value compared to previous study. Carrier concentrations were calculated via$n = \frac{IB}{eV_H d}$, in which I is the current, B is the magnetic field, V_H is Hall voltage, d is the film thickness.

As the film crystalline degree decrease, V_H decrease with anomalous carrier concentration n_{mc} = 2*10^{21} cm^{-3} for MC sample and V_H almost quench in the PC samples, which could be attributed to the suppression of the carrier's 2D transport with the weakening of the intermolecular couplings, the carrier localized within in the one-dimensional polymer chain could hardly realize normal Lorentz deflection under magnetic field.

Utilizing the differences in the dependence on the sign of the magnet field, we extract Hall voltage as shown in the right, by V_{HALL}~0.5*(V(+B)-V(-B)) (green circles)or by the method similar with that in Ref. 8 (red circles).

Part. 5b

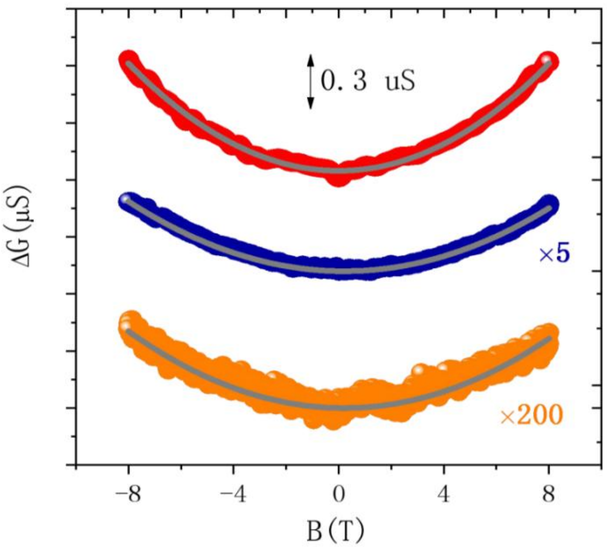

Supplementary Figure 5b. Magnetic field *B* dependence of magneto conductance (Δ G(B) = G(B) − G(0)) for three kinds of samples. The fitting curves are based on the 2D Hikami-Larkin-Nagaoka model $\Delta G(B) = \frac{1}{24}\frac{e^2}{2\pi^2\hbar}\frac{B^2}{B_\varphi^2}$, where $B_\varphi = \frac{\hbar c}{4eD\tau}$ the fitting suggests the weak localization effect contributed by the 2D coherent carriers. The inelastic scattering length is extracted with$\lambda = \sqrt{D\tau}$, values of λ reach 5.6 nm and 2.9 nm for HC and MC samples, while for the PC samples, the values are around 1 nm, comparable to the intermolecular lattice constant, which could be attributed to the lack of 2D carriers.

Part. 6

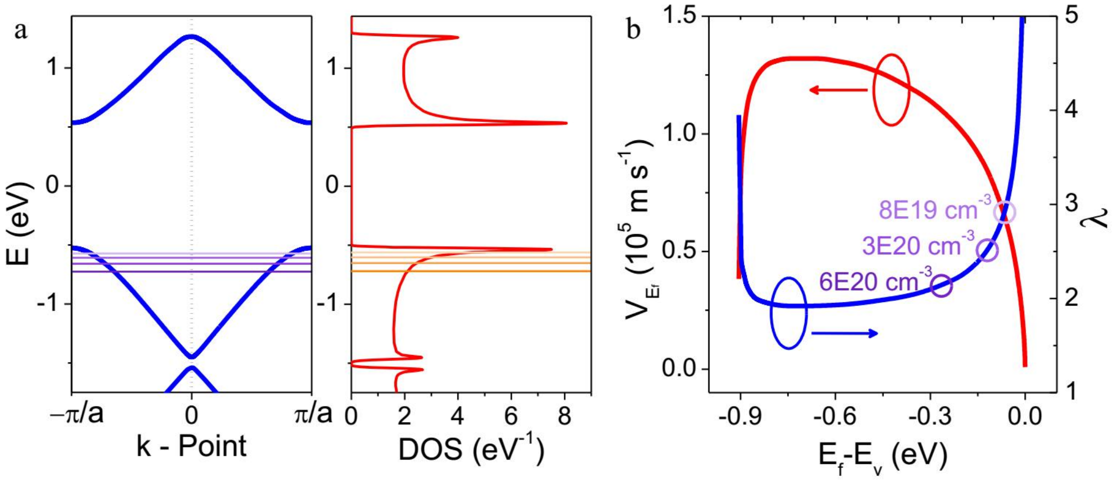

Supplementary Figure 6. Theoretic evaluation of the power-law nature in the 1D polymer chain by first-principle calculations with 1D Bosonization approach. a. Band dispersion and density of states (DOS) of PBTTT 1D polymer chain, the position of Fermi level was determined with various holes' density on one repeat unit, ranging from 0.1 to 0.7. **b**. The energy-dependent Fermi velocity (red line), and the relevant power-law exponent (blue line) derived with the Bosonization model, different concentrations of carriers are marked on the curves by circles.

Specially, to gain a further insight into the polymer chains' 1D electronic liquid characters, we investigated the molecule's electronic structure and the relevant power-law characters by first-principle calculations, and significantly together with the developed Bosonization method for quasi-1D Fermions systems [9,10]. The calculated band dispersion and density of states (DOS) for the polymer 1D chain are displayed in Supplementary Figure 6a, the Fermi level could be determined with certain hole density per unit in the polymer chain. The Fermi velocity V_{Ef} is obtained at the knowledge of the *E-K* relation and Fermi level. We obtain the carrier density dependent power-law exponent λ, With Eq (S1) from the Bosonization approach [11].

$$\lambda = \frac{1}{2}\left[\frac{1}{2\sqrt{\frac{4\pi g}{v_{E_f}}+1}} + \frac{1}{2}\sqrt{\frac{4\pi g}{v_{E_f}}+1}\right] - \frac{1}{2}, \qquad \text{(S1)}$$

in which, $g = \frac{\tilde{V}(0)}{2\pi^2}$ is the electrons' correlation strength, $\tilde{V}(0) = 2(e^2/\varepsilon)Ln(L/R)$ is the scattering element, the parameter ε, *L*, and *R* stand for the dielectric constant, 1D chain's length, and the 1D electrons average radius, respectively [9, 11]. For *L/R* have the similar values for

PBTTT and the single wall nanotube (SWNT) [12], and values of ε do not vary much for carbon materials, we take the scattering element value of SWNT reported for universal reference, where $\widetilde{V}(0) = 7.47\pi v_{SWNT}$, $v_{SWNT} = 8.0\times10^5$ m/s is the Fermi velocity of SWNT. As shown in Supplementary Figure 6b, the power-law exponent' values almost keep unchanged around $\alpha = 2$ at large carrier density, while increase dramatically as carrier density decreases. The $\alpha - E$ curve is expressly marked at carrier concentrations $n = 8 \times 10^{19}$, 3×10^{20}, and 6×10^{20} cm^{-3}, with the power-law exponent values $\alpha = 2.95$, 2.5, and 2.1, respectively.

Part. 7

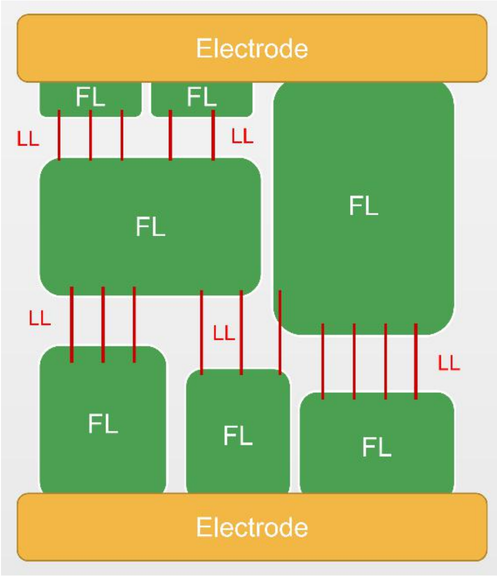

Supplementary Figure 7. Schematic diagram of the equivalent circuit of the Fermi liquid - Luttinger liquid resistive network.

We assume that the resistance of FL-LL tunneling is far larger than that of charge transport in FL regions ($R_{FL\text{-}LL}$>>R_{FL}, R_{FL}=0). When the FL-LL tunneling junction features by the LL transport behaviors, $I{\sim}V^{\beta}$, $G{\sim}T^{\alpha}$, the whole film could also obey the relationship. There are in total *m* parallel paths in the charge transport channel, and *n* tunneling junctions in one path, thus we have

$$I_m = a_{n,m} {V_{n,m}}^{\beta}$$

$$V_{n,m} = \left(\frac{1}{a_{n,m}}\right)^{1/\beta} {I_m}^{1/\beta}$$

$$U = \sum_n V_{n,m} = \sum_n \left(\frac{1}{a_{n,m}}\right)^{1/\beta} {I_m}^{1/\beta}$$

$$I_m = \left(1/\sum_n \left(\frac{1}{a_{n,m}}\right)^{1/\beta}\right)^{\beta} U^{\beta}$$

$$I = \sum_m I_m = \sum_m \left(1/\sum_n \left(\frac{1}{a_{n,m}}\right)^{1/\beta}\right)^{\beta} U^{\beta} \rightarrow\rightarrow I{\sim}U^{\beta}.$$

We have $I{\sim}U^{\beta}$. Similarly, we have $G{\sim}T^{\alpha}$.

Part. 8

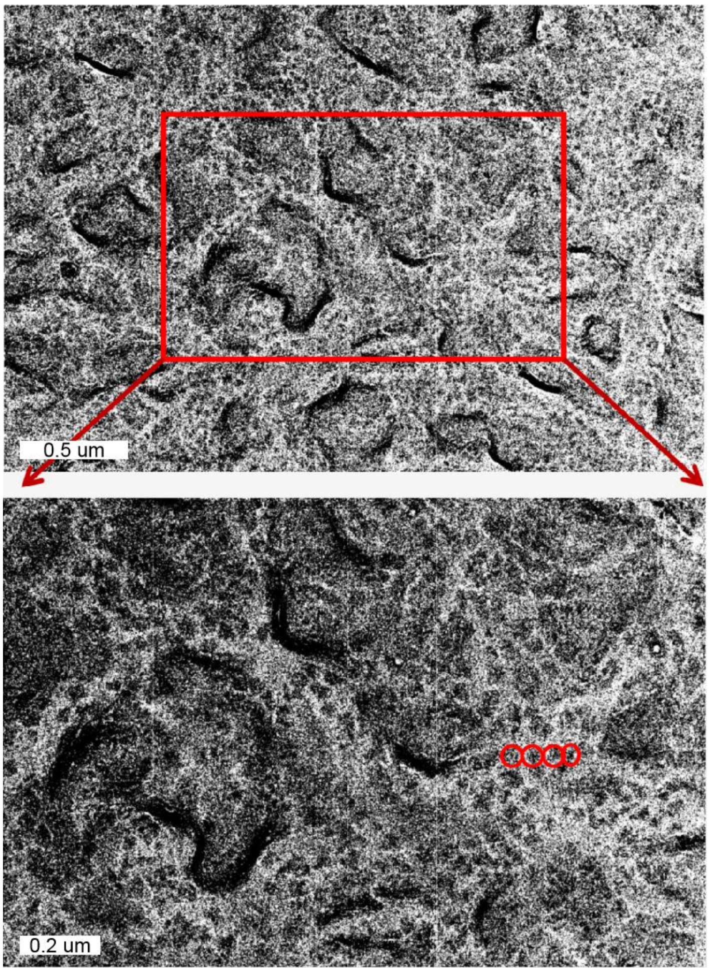

Supplementary Figure 8. TEM image of surface morphology of HC PBTTT film. The PBTTT thin films for TEM characterization were prepared via transfer method. The bared copper grids *without any polymer supporting layer* were used. The difference of electron beam transmission intensity in crystalline and amorphous regions could reveal the distribution of crystalline grains, as shown by the dark sites, four dark sites are marked by red circles. The scale bar is 0.5 um, 0.2 um at the upper panel and the low panel, respectively.

Part. 9

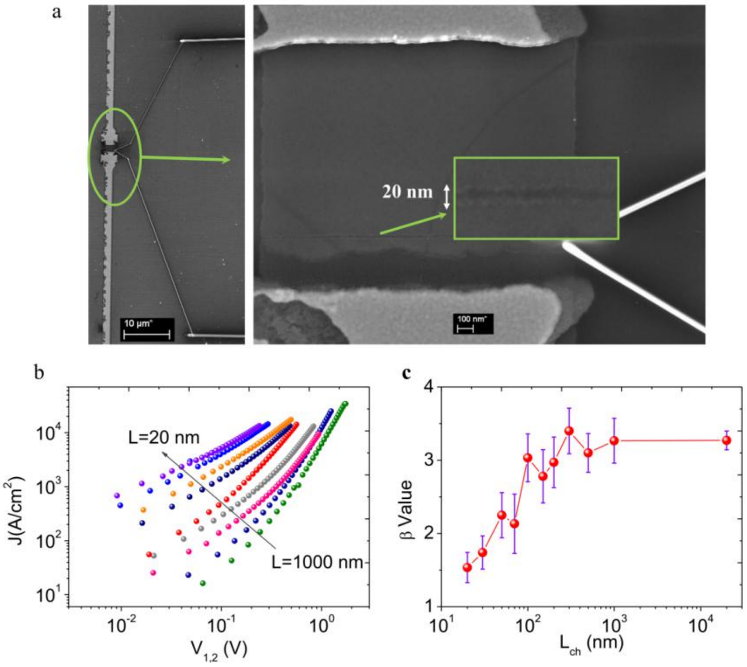

Supplementary Figure 9. Submicrometer channel device for nonlinear transport investigations. a. SEM images of the device with short channel length L_{ch} =20 nm, the scale bars are 10 um (left) and 100 nm (right), respectively. **b**. *I-V* curves of the devices with various channel lengths ranging from 1000 nm down to 20 nm, displayed in log-log coordinates. **c**. The β values extracted from the devices of various channel lengths.

Based on our mesoscopic model, the resistance in the large crystalline grains, which are treated as Fermi liquids, should be Ohmic. Consequently, if we narrow the channel length to around or even smaller than the grains' character size, there is in large probability that one transport path is occupied by a single FL, thus the *I-V* curves should behave likes Ohmic contact. And this assumption was certified with electrical characterization on HC samples with various channel lengths from 300 to 20 nm. Supplementary Figure 9a shows the SEM images of the device's short channel of length L_{ch} = 20 nm at different zoom scales. As shown in Supplementary Figure 9b, the nonlinearity of *I-V* curves are evidently suppressed with the channel shortening to below 100 nm, the extracted values of power-law exponent β shown in Supplementary Figure 9c decreased from more than 3 to around 1.5 indicates the crossover to Ohmic transport, the obvious phenomenon are consistent well with our mesoscopic model of FLs and LLs. We employed patterned graphene as electrode to ensure the molecular packing order degree at the contact edge.

The graphene electrode's thickness is less than 1 nm. While the conventional metal contact always feature of tens nanometers, which would induce larger disorders in the polymer and affect the grain nucleation in such a short channel.

Part. 10

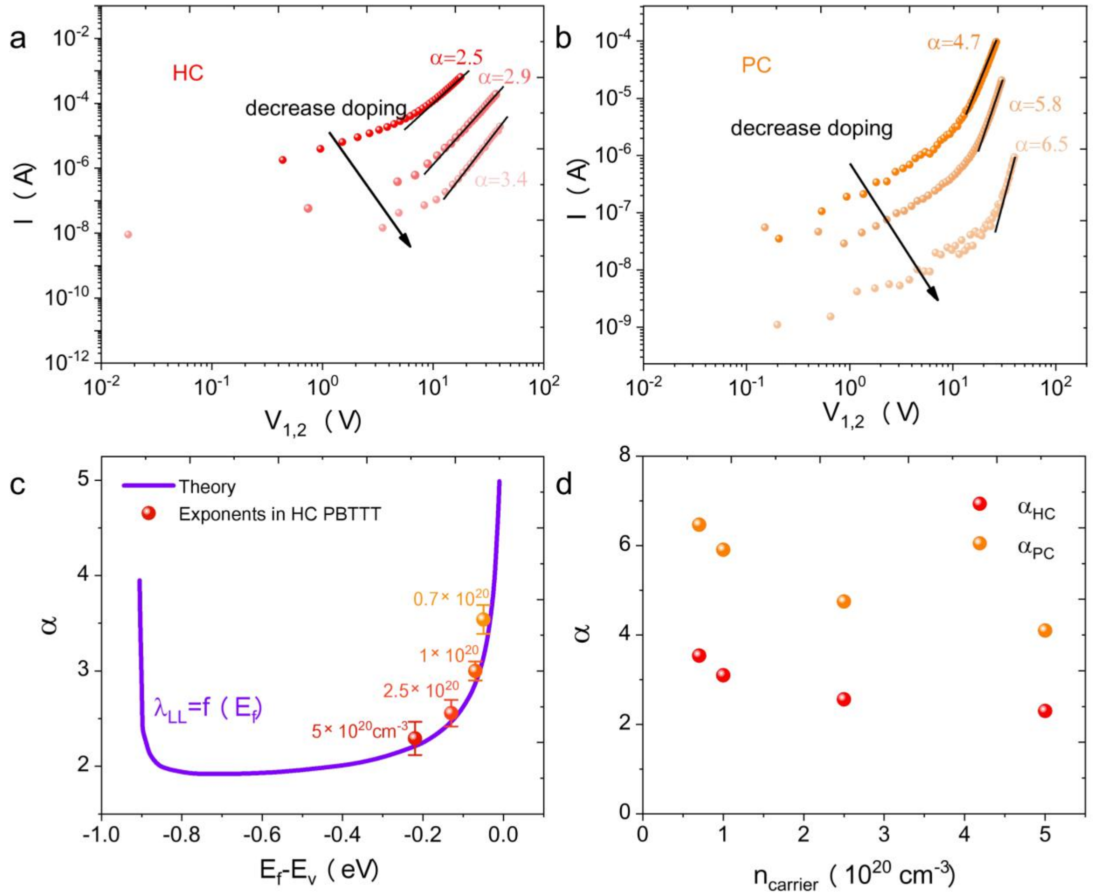

Supplementary Figure 10. Carrier density dependent power-law behavior in HC and PC PBTTT samples. *I-V* curves at 5.5 K for HC (a) and PC (b) samples at various carriers' concentrations. c. Extracted power-law exponents in HC PBTTT samples at various carriers' concentrations (n, cm^{-3}), compared with the theoretic curves. d. Comparison between the values of power-law exponents for HC and PC samples at various carriers' concentrations (n)， relation between α_{HC} and α_{PC} still approximately obey the underlying relationship of $\alpha_{LL\text{-}LL} = 2\alpha_{FL\text{-}LL}$.

Part.11

Supplementary Table 1. Grain size, grain density, and diffraction ring's HWHM of three type samples.

Samples	Grain size(nm)	Grain density(um^{-2})	[010] Ring HWHM (nm^{-1})
HC	30~40	300~400	0.12~0.15
MC	17~25	100~200	0.21~0.29
PC	~10	~50	>0.37

From the statistical data of grains, the HRN picture displayed in Fig.3 could be validated to some degree. We are inferred from the HC sample's data that every grain has 2D space of 2500 nm^2, the grain size is about 30~40 nm, this means that there is 10~20 nm intergrain space for tie-chains' formation, which is reasonable for the distribution of large amount of FL-LL tunneling junctions. While for the MC samples, the intergrain space reaches larger than 50 nm, thus tie-chains form by chance, FL-LL tunneling and LL-LL tunneling coexist. When it comes to PC samples, grains are too small and the distribution is too sparse for tie-chains' formation, thus only tunneling among weakly coupled 1D chains, i.e. LL-LL tunneling exists.

Part.12

Supplementary Table 2. Statistical values of conductivity σ and power-law exponents α for doped P3HT and IDT-BT.

P_3HT Samples	#1	#2	#3	#4	#5	#6	#7
σ	8.1	7.2	4.9	4.7	1.3	0.9	1.4
α	1.61	1.55	1.52	1.71	3.31	3.0	2.74

IDT-BT Samples	#1	#2	#3	#4
σ	9.4	1.1	3.2	1.8
α	2.45	2.38	2.29	2.44

Details for sample preparations:

HC samples (#1-4) based on P3HT, spin coating of P3HT's CB solution (3mg/mL) onto OTS treated SiO_2 substrate, with 140 °C annealing post-coating treatment.

PC samples (#5-7) based on P3HT, spin coating of P3HT's chloroform solution (12mg/mL) onto non-modified SiO_2 substrate etched by HF gas, with surface roughness as high as 6~10 nm, with no annealing process.

IDT-BT: #1 drop casting (0.5 mg/ml, chloroform solution)

#2 spin coating (5 mg/ml, chloroform solution)

#3 off center spin coating, spin radical direction is along the channel (2mg/ml, chloroform solution)

#4 off center spin coating, spin radical direction is vertical with the channel (2mg/ml, chloroform solution)

Part. 13

Supplementary Note 1: We choose the concept 'Fermi liquid' to depict the ordered grain in crystalline polymer, instead of 'Fermi gas' in traditional semiconductor. It's due to the much larger carrier concentration in the conducting polymer used in our work, which reached more than 10^{20} cm^{-3}, while those in traditionally operated transistors whole could be described as Fermi gas are only around 10^{18} cm^{-3}. In this work, the crystalline grains behave like metals at low temperature, which are systems of interacting Fermions, and should be treated as Fermi liquid. What should be noted is, the interaction of electrons in Fermi liquid hardly change the excitation behavior by electrical field, thus Fermi liquid also exhibit linear Ohmic *I-V* relations as those of Fermi gas.

Part. 14

Supplementary Note 2: We investigated the nonlinear transport mainly in low temperature, for the molecular vibration would induce dynamical disorder in the system, which would deteriorate the quantum liquid scenario and the system might turn from liquid to Fermi glass. For example, the intrachain torsion for polymer would break the coherence of electron, and therefore, the Luttinger liquid phase might fail to describe the 1D polymer chains. Experimentally, we observed deviation of *I-V* data from the universal scaling in different polymer samples, as shown in the Supplementary Figure 11.

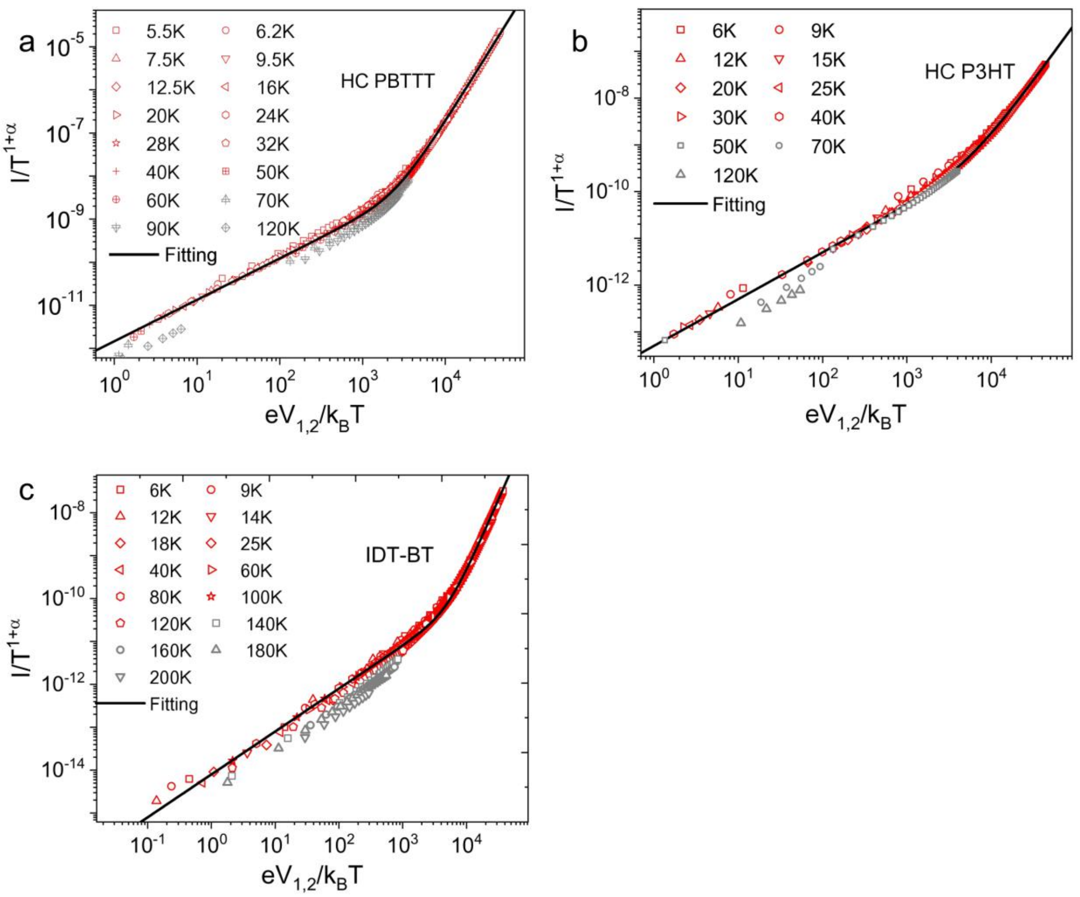

Supplementary Figure 11 Universal scaling of three types of polymers at wider temperature range.

The polymer IDT-BT features by more rigid backbone of the 1D chain, which could suppress the dynamical disorder to some degree, this might lead to the relative higher deviation temperature up to around 140 K in IDT-BT, while this temperature is evidently lower in PBTTT (70 K) and P3HT (50 K).

Supplementary References:

[1] Rodin, AS. et al., Apparent Power-Law Behavior of Conductance in Disordered Quasi-One-Dimensional Systems. Phys. Rev. Lett., 105, 106801 (2010)

[2] Grabert, H. et al., Quantum tunnelling rates for asymmetric double-well systems with Ohmic dissioation. Phys. Rev. Lett. 54, 1605–1608 (1985)

[3] Fisher, M. et al., Dissipative quantum tunnelling in a biased double-well system at finite temperatures. Phys. Rev. Lett. 54, 1609–1612 (1985)

[4] Akai-Kasaya, M, et al., Coulomb Blockade in a Two-Dimensional Conductive Polymer Monolayer. Phys. Rev. Lett., 115, 196801 (2015)

[5] Abdalla, H. et al., Effective Temperature and Universal Conductivity Scaling in Organic Semiconductors. Sci. Rep., 5, 16870 (2015)

[6] Kaiser, AB. et al., Electronic transport properties of conducting polymers and carbon nanotubes. REP PROG PHYS, 64, 1-49 (2001)

[7] Kang, E. et al., Multi-barrier field-emission behavior in PBTTT thin films at low temperatures. Sci. Rep., 5, 8396 (2005)

[8] Kang, K. et al. 2D coherent charge transport in highly ordered conducting polymers doped by solid state diffusion. Nat. Mater. 15, 896 (2016).

[9]Yoshioka, H. et al., Tomonaga-Luttinger liquid theory for metallic fullurene polymers. Phys. Rev. B, 93, 165431 (2016)

[10]Yoshioka, H. et al., Density of states anomalies in multichannel quantum wires. Phys. Rev. B, 84, 075443 (2011)

[11]Egger, R., Luttinger Liquid Behavior in Multiwall Carbon Nanotubes. Phys. Rev. Lett., 83, 5547 (1999)

[12] Ishii, H., Direct observation of Tomonaga–Luttinger-liquid state in carbon nanotubes at low temperatures. Nature, 426, 540 (2003)
